# Multidomain Predictors of Four-Year Risk for Dementia and Mild Cognitive Impairment Among Community-Dwelling Korean Older Adults

**DOI:** 10.3390/healthcare14020184

**Published:** 2026-01-12

**Authors:** Jinhee Shin, Hyebeen Sim, Kennedy Diema Konlan, Chang Gi Park

**Affiliations:** 1 College of Nursing, Woosuk University, Wanju 55338, Republic of Korea; jhshin@woosuk.ac.kr; 2 College of Nursing, University of Illinois Chicago, Chicago, IL 60612, USA; parkcg@uic.edu; 3 Division of Nursing, Kangbuk Samsung Hospital, Seoul 03181, Republic of Korea; 4 Department of Public Health Nursing, School of Nursing and Midwifery, University of Health and Allied Sciences, Ho PMB 31, Ghana; dkkonlan@uhas.edu.gh

**Keywords:** dementia, mild cognitive impairment, aging, multimorbidity, multinomial logistic regression, Korean Longitudinal Study of Aging (KLoSA), social interaction, Republic of Korea

## Abstract

Background: Dementia and mild cognitive impairment (MCI) are major public health concerns in rapidly aging societies. However, evidence from non-Western populations with self- or proxy-reported physician-diagnosed cognitive outcomes remains limited. Methods: This study investigated the four-year (2018–2022) incidence of self- or proxy-reported dementia and MCI, and their multidomain risk markers in a nationally representative sample of 3432 Korean adults aged ≥ 65 years from the Korean Longitudinal Study of Aging. Weighted descriptive analyses and multinomial logistic regression accounting for the complex survey design (sampling weights, strata, and clusters) were used to identify demographic, functional, mental health, and social factors associated with the incidence of cognitive impairment. Adjusted predicted probabilities were estimated for age, depressive symptoms, and instrumental activities of daily living (IADL). Results: Over the four-year follow-up period, among 3432 initially cognitively normal community-dwelling older adults, 34 participants developed MCI and 70 developed dementia. Older age, increased IADL impairment, depressive symptoms, and multimorbidity were significantly associated with a higher likelihood of incident cognitive impairment, while living with family was associated with a lower likelihood. Frequent social interaction showed a protective association against dementia. Predicted probabilities demonstrated overall increasing trends across age, depressive symptoms, and IADL, reflecting general risk patterns. Conclusions: These findings emphasize the importance of multidomain assessments for early detection and community-based prevention strategies. By identifying key clinical and social markers, this study provides culturally relevant evidence to support dementia risk management in rapidly aging populations, highlighting the protective roles of family co-residence and frequent social interaction among Korean older adults.

## 1. Introduction

Cognitive health is a core component of healthy aging and late-life functioning [1,2]. Cognitive aging is heterogeneous, with substantial interindividual variation in late-life trajectories [3]. Dementia is an example of cognitive impairment that is reported to affect over 55 million adults worldwide, with an estimated economic burden exceeding 1.1% of global gross domestic product [4,5]. Cognition plays a critical role in aging, as global memory loss is usually associated with brain atrophy, leading to an increased risk of dementia and related unwarranted influence on mental well-being [6,7]. This cognitive impairment may result in adverse health consequences [3] and is typically progressive, leading to substantial functional decline [8]. The progression of cognitive impairment often involves an intermediate stage, usually referred to as mild cognitive impairment (MCI), in which cognitive decline is present without substantial impairment in activities of daily living. Consequently, recognizing MCI as a crucial transitional stage is essential for the early detection and prevention of subsequent dementia [9].

Identifying modifiable multidomain factors that contribute to the incidence of MCI and dementia in community-dwelling older adults is essential for guiding risk assessment and developing scalable, community-based, culturally sensitive, and acceptable prevention strategies [8,10]. More frequent social contact has been consistently associated with a lower risk of incident MCI and dementia [11]. In addition, multidomain lifestyle interventions may slow cognitive decline, underscoring the potential scalability of prevention strategies [10].

Cognitive impairment outcomes are affected by the interaction of demographic and socioeconomic factors (such as older age and lower education) [12], health conditions (including multimorbidity, sensory impairments, and functional limitations like instrumental activities of daily living [IADL]) [13,14,15], mental health issues (such as depressive symptoms) [16], and health behaviors (including smoking, alcohol use, and physical activity) [8,17]. In addition, social factors, including social interaction and isolation, are also important correlates of cognitive health in older adults [11,18].

Against this backdrop, given the substantial consequences of dementia for older adults’ development and functioning, there is a growing need to identify predictors of cognitive impairment to inform intervention strategies using existing population-based data. Despite this need for comprehensive evidence, important research gaps remain, particularly with respect to multidomain predictors in non-Western populations [19]. This study addresses these gaps in several ways. First, few population-based studies in Asian settings have examined the incidence of MCI and dementia using self- or proxy-reported physician-diagnosed outcomes. Second, recent cohort studies do not necessarily account for secular changes in education, health behaviors, and the prevalence of multimorbidity [8]. Third, recent studies have primarily reported adjusted associations; however, the presentation of adjusted predicted probabilities across meaningful ranges of key risk factors may enhance clinical interpretability and support individualized risk assessment in clinical and community settings.

This study is explicitly situated within the biopsychosocial model [20], which conceptualizes health outcomes as arising from the interaction of biological, psychological, and social processes. This theoretical perspective is further aligned with the World Health Organization’s healthy ageing framework [21], which operationalizes these interacting domains in later life by emphasizing intrinsic capacity—including cognitive, psychological, and physical functions—and its dynamic interaction with social and environmental contexts. Together, these established frameworks provide a coherent basis for conceptualizing cognitive decline as a multidimensional process shaped by demographic vulnerability, chronic disease burden, functional limitations, mental health, and social engagement in older adulthood.

Using data from the Korean Longitudinal Study of Aging (KLoSA), a nationally representative cohort of community-dwelling older adults, this study examined multidomain risk markers and early indicators, rather than causal determinants, of four-year incident MCI and dementia. Although MCI and dementia are often conceptualized along a continuum of cognitive decline, they represent clinically and prognostically distinct conditions. MCI is characterized by objective cognitive decline with preserved functional independence, whereas dementia involves substantial impairment in daily functioning and loss of independence. Importantly, not all individuals with MCI progress to dementia, and risk markers may differ in their relevance and strength across these stages. Accordingly, the concurrent examination of incident MCI and dementia allows for the identification of both shared multidomain risk markers and stage-specific patterns, which are clinically meaningful for early detection, risk stratification, and targeted prevention strategies. In addition, South Korea represents a critical context for such investigation, given its rapid population aging and distinctive sociocultural emphasis on family co-residence, which renders social and household factors—such as living with family—particularly salient for understanding cognitive health trajectories [22,23]. By adopting this multidomain and theory-informed approach, the present study provides practical evidence to inform clinical screening and community-based prevention strategies in rapidly aging societies.

## 2. Materials and Methods

### 2.1. Data Source and Participants

The Korean Longitudinal Study of Aging (KLoSA) is a nationally representative ongoing panel of community-dwelling Koreans aged 45 years and older. Initiated in 2006 by the Korea Labor Institute, KLoSA follows respondents biennially with a consistent core questionnaire and periodically updates the sample to maintain representativeness as cohorts age. Participants are chosen through a multistage, stratified probability sampling process, and trained interviewers conduct face-to-face, computer-assisted personal interviews. The survey includes sociodemographic and family characteristics, health status and behaviors, employment, income and expenditures, assets, and subjective expectations. Wave 7 (2018) of KLoSA served as the baseline, and incident cognitive outcomes were ascertained at Wave 9 (2022).

#### Population and Sample

**Figure 1:**
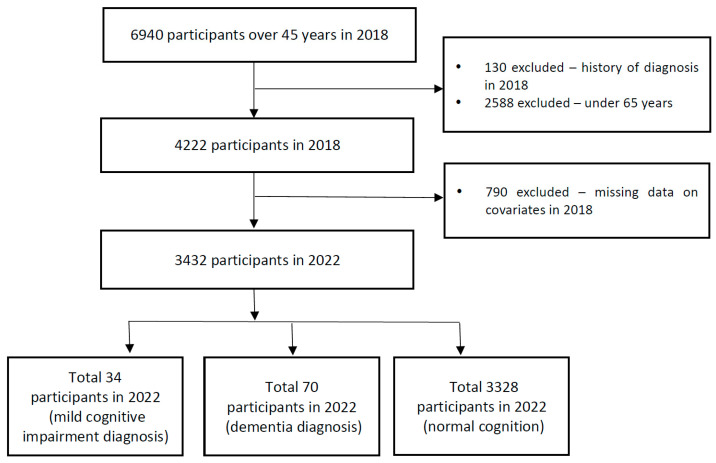
Participant selection process in the Korean Longitudinal Study of Aging.

Among the 6940 respondents aged ≥ 45 years who participated in the 2018 wave, those aged < 65 years (n = 2588) and those with prevalent physician-diagnosed cognitive impairment at baseline (n = 130) were excluded, leaving 4222 eligible participants. Participants with missing baseline covariates (n = 790) were then excluded, resulting in a final analytic sample of 3432 individuals with complete 2018 data and cognitive outcomes ascertained in 2022 (Figure 1). At follow-up (2022), participants were classified as having normal cognition (n = 3328), MCI (n = 34), or dementia (n = 70). KLoSA public-use files are de-identified and are available via the Korea Labor Institute data portal.

### 2.2. Measures and Variables

#### 2.2.1. Incident Cognitive Outcomes

Incident cognitive outcomes between 2018 and 2022 were defined based on self- or proxy-reported physician diagnosis. At the 2022 wave, respondents (or their proxies) reported whether a clinician had ever informed them of a diagnosis of MCI or dementia (e.g., “Have you been diagnosed with MCI or dementia by a physician?”; yes or no). In South Korea, physician diagnosis of MCI or dementia is made within a national dementia care framework and is required for access to dementia-related healthcare services and benefits; such diagnoses are typically informed by internationally accepted clinical criteria, including DSM-based and NIA-AA guidelines for dementia and Petersen-type criteria for MCI. A three-category, mutually exclusive outcome was defined: normal cognition (no self- or proxy-reported physician diagnosis), MCI, and dementia.

#### 2.2.2. Demographic Characteristics

Demographic variables included age (years; continuous); gender (male or female); education level (elementary school or less, middle school, high school, or college or higher); living arrangement (living alone or living with family); religion (yes or no); and household income in the prior year (continuous).

#### 2.2.3. Health Status and Health Behaviors

Health status variables included body mass index (BMI; kg/m^2^; continuous); handgrip strength (kg; range 0–50; average of right and left hands); number of chronic diseases (count, range 0–9), defined as self-reported physician-diagnosed hypertension, diabetes, cancer, arthritis/rheumatism, chronic lung disease, liver disease, heart disease, cerebrovascular disease, or psychiatric illness; vision impairment and hearing loss (yes or no), defined as self-reported difficulty performing daily activities due to vision or hearing problems; IADL score (Korean IADL; 10 items scored 1 = independent, 3 = partially dependent, 5 = completely dependent; total range 10–50, with higher scores indicating greater functional limitation) [24]; and depressive symptoms measured by the Korean 10-item Center for Epidemiologic Studies Depression scale (CES-D10; range 0–30; higher scores indicate more symptoms) [25]. Health behaviors included smoking status (never, past, current), alcohol consumption (current, past, never), and regular exercise (yes or no; at least once per week).

#### 2.2.4. Social Interactions

The frequency of face-to-face contact social interactions with close others (friends, relatives, acquaintances) was measured using a 10-point ordinal item, with higher values indicating more frequent contact. Response options were: 1 = no close contacts; 2 = almost never; 3 = once or twice per year; 4 = three to four times per year (about once every 3–4 months); 5 = five to six times per year (about once every two months); 6 = about once per month; 7 = about twice per month (about every two weeks); 8 = about once per week; 9 = two to three times per week; 10 = almost daily (≥4 times per week). For primary analysis, this 1–10 score was treated as a continuous predictor to preserve the full range of information and maximize statistical power. While this measure is ordinal, treating it as a continuous variable is common practice for ordinal scales with multiple response categories and has been shown to yield robust and interpretable results in regression analyses [26].

### 2.3. Data Analyses

Descriptive analyses were performed accounting for the complex, multistage survey design of KLoSA, including sampling weights, strata, and primary sampling units. Baseline characteristics were summarized as means (standard deviations) or counts (percentages) and compared across 2022 cognition outcome categories (normal, MCI, dementia) using analysis of variance (ANOVA) for continuous variables and χ^2^ tests for categorical variables. To identify predictors of incident MCI and dementia, a survey-weighted multinomial logistic regression model was fitted, with normal cognition as the reference outcome, and relative risk ratios (RRRs) with *p*-values were reported. Given the relatively small number of incident cases, particularly for MCI, sensitivity analyses using a binary outcome (any cognitive impairment [MCI or dementia] vs. normal cognition) were conducted. At the same time, multinomial logistic regression was retained to allow separate examination of MCI and dementia as clinically distinct cognitive states, given the recognized clinical and prognostic relevance of MCI as an intermediate stage of cognitive decline. Continuous predictors were modeled assuming linear effects on the log-odds scale, consistent with standard practice in population-based epidemiologic analyses.

Adjusted predicted probabilities were subsequently derived via marginal standardization from the survey-weighted multinomial models, with continuous covariates fixed at their sample means and categorical variables set to reference categories. These probabilities were visualized across age, depressive symptoms (CES-D10), and instrumental activities of daily living (IADL) [27]. Multinomial logistic regression was selected because cognitive status was assessed at discrete survey waves, and transitions between cognitive states could not be assumed to follow a strictly linear or temporally ordered process, thereby limiting the appropriateness of ordinal or time-to-event modeling approaches. Model adequacy was assessed using the likelihood-ratio χ^2^ test and McFadden pseudo-R^2^; multicollinearity was examined using variance inflation factors (VIFs). A two-sided *p* < 0.05 was considered statistically significant. Analyses were conducted using Stata/SE 17 (StataCorp, College Station, TX, USA).

### 2.4. Ethical Considerations

The data in this study were collected with authorization from Statistics Korea (Approval No. 33602). All participants provided informed consent through a standardized survey process before taking part in the computer-assisted personal interview, in accordance with the Statistics Act and the Declaration of Helsinki. The de-identified KLoSA datasets are publicly available for scientific research. This secondary analysis was reviewed and approved as exempt by the Institutional Review Board of Woosuk University, as it used publicly available, de-identified data (IRB No. WS-2025-28).

## 3. Results

### 3.1. General Characteristics

A total of 3432 community-dwelling older adults were included at the 2018 baseline. By the 2022 follow-up, 34 participants had developed MCI, and 70 had developed dementia, while 3328 remained cognitively normal. Table 1 presents baseline characteristics by cognitive outcome status.

The mean age of the sample was 74.4 years, and 59.0% were female. More than half (52.1%) had completed elementary school or less, and most participants lived with family members (79.8%). Regarding health behaviors, 53.7% were non-drinkers, 70.8% were non-smokers, and 34.3% engaged in regular exercise.

Across cognitive outcome groups, individuals who later developed MCI or dementia were older and had lower educational attainment than cognitively normal participants. They also had lower BMI, reduced handgrip strength, and a greater number of chronic diseases. In addition, those with incident MCI or dementia reported higher depressive symptoms and greater IADL limitations, along with markedly lower social interaction frequencies.

Significant group differences were observed in age, education, living arrangement, household income, BMI, handgrip strength, number of chronic diseases, depressive symptoms (CES-D10), IADL scores, social interaction frequency, and regular exercise (all *p* < 0.05). No significant differences were observed for sex, religion, alcohol consumption, smoking status, hearing aid use, or vision problems.

### 3.2. Multivariable Predictors of Incident Cognitive Outcomes

Table 2 presents adjusted relative risk ratios (RRRs) from the multinomial logistic regression with normal cognition as the reference outcome. Older age was associated with a higher risk of both outcomes (MCI: RRR = 1.10, *p* = 0.014; dementia: RRR = 1.12, *p* < 0.001). Compared with living alone, living with family was associated with a lower risk of MCI (RRR = 0.31, *p* = 0.023) and dementia (RRR = 0.28, *p* = 0.003). This corresponds to an approximately 69% lower relative risk of incident MCI and a 72% lower relative risk of incident dementia, respectively, among older adults living with family compared with those living alone. A greater number of chronic diseases was associated with increased dementia risk (RRR = 1.48, *p* = 0.008). Depressive symptoms (CES-D10) were positively associated with both MCI (RRR = 1.09, *p* = 0.032) and dementia (RRR = 1.24, *p* < 0.001). Higher IADL impairment was associated with increased risk of MCI (RRR = 1.11, *p* < 0.001) and dementia (RRR = 1.07, *p* = 0.012). Social interactions were inversely associated with dementia (RRR = 0.72, *p* < 0.001), whereas the association with MCI was not statistically significant (RRR = 0.97, *p* = 0.741). Model fit was adequate (LR χ^2^(42) = 288.53, *p* < 0.001; McFadden pseudo-R^2^ = 0.385), and multicollinearity was acceptable (maximum VIF = 3.45). To address potential instability due to low events-per-variable (EPV) in the MCI group (n = 34), we conducted a sensitivity analysis using a binary outcome combining incident MCI and dementia (any cognitive impairment) versus normal cognition. The associations of age, IADL impairment, depressive symptoms, multimorbidity, living with family, and social interaction were directionally and statistically consistent with the primary multinomial models, supporting the robustness of the main findings.

### 3.3. Average Adjusted Probabilities of Incident Cognitive Outcomes

**Figure 2:**
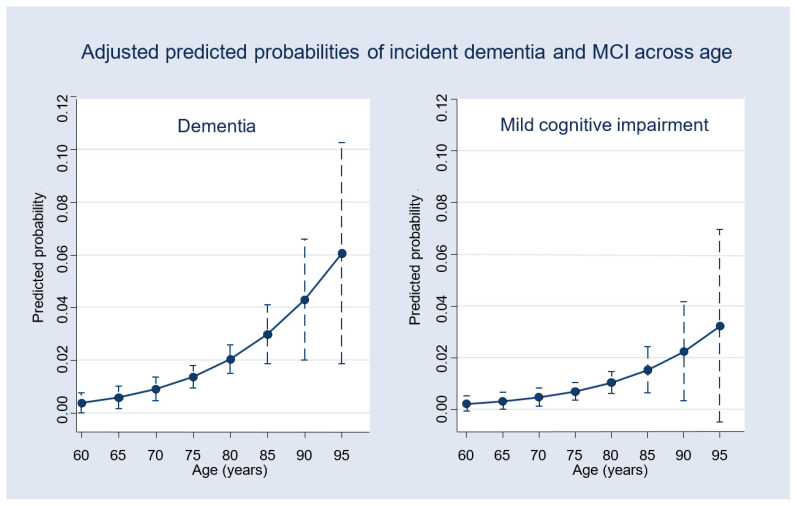
Adjusted predicted probabilities of 4-year incident dementia (**left**) and MCI (**right**) across age, derived from the multinomial logistic regression model with all other covariates held at their mean values.

**Figure 3:**
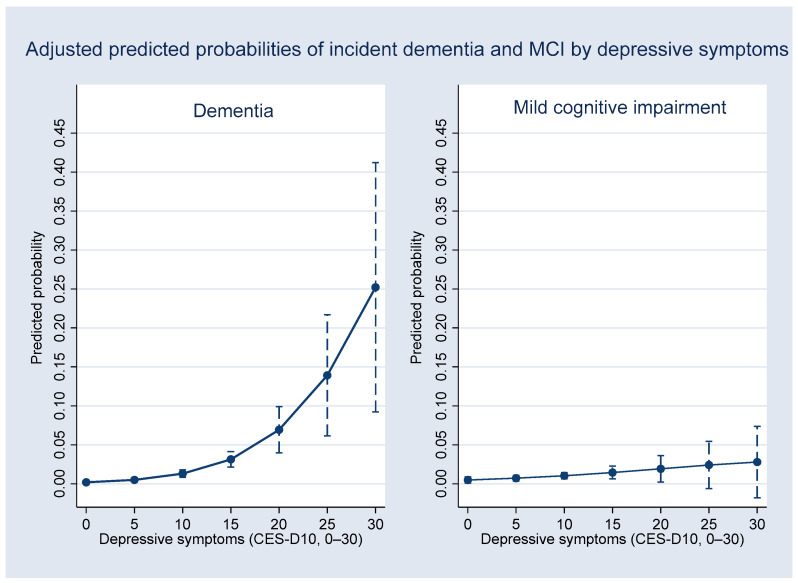
Adjusted predicted probabilities of 4-year incident dementia (**left**) and MCI (**right**) across depressive symptom severity (CES-D10), derived from the multinomial logistic regression model with all other covariates held at their mean values.

**Figure 4:**
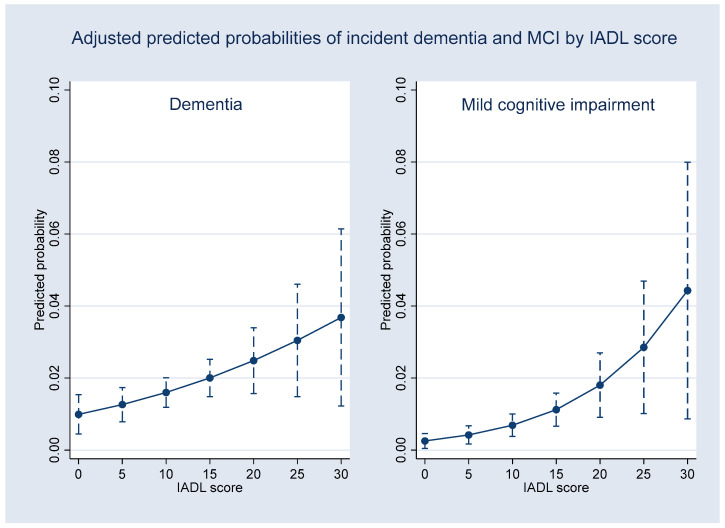
Adjusted predicted probabilities of 4-year incident dementia (**left**) and MCI (**right**) across instrumental activities of daily living (IADL) score, derived from the multinomial logistic regression model with all other covariates held at their mean values.

Figure 2, Figure 3 and Figure 4 illustrate the adjusted predicted probabilities of 4-year incident MCI and dementia based on the multinomial logistic regression model (reference category: normal cognition). As shown in Figure 2, the predicted probability of dementia generally increased with age, suggesting an overall upward trend, whereas the probability of MCI increased more gradually. Figure 3 displays the predicted probabilities across levels of depressive symptoms (CES-D10), showing higher probabilities of both MCI and dementia at higher symptom levels, with increasing uncertainty at the upper range of CES-D10 scores, as reflected by wider confidence intervals. Figure 4 shows the predicted probabilities by IADL impairment. Greater functional limitations were associated with a substantially higher probability of MCI and a comparatively smaller but still measurable increase in dementia probability. Across all figures, predicted probabilities illustrate general risk patterns across key predictors.

## 4. Discussion

This study identified critical multidomain predictors of four-year MCI and dementia in a nationally representative cohort of community-dwelling Korean older adults. Importantly, these findings suggest that incident cognitive impairment arises from the concurrence of vulnerabilities across demographic, functional, mental health, and social domains, rather than from isolated risk factors. By simultaneously modeling multiple domains, this study highlights the multifactorial and interdependent etiology of late-life cognitive decline, reflecting real-world clinical and community settings in which risks accumulate across domains. Accordingly, a multidomain framework offers a more comprehensive basis for prevention strategies than single-domain approaches.

The results indicate that adjusted predicted probability analyses were used to contextualize these associations by illustrating general risk patterns across age, depressive symptoms, and IADL, rather than precise, linear, or deterministic dose–response relationships. Notably, the steeper gradient observed between IADL impairment and MCI compared with dementia (as shown in Figure 4) warrants careful interpretation. This pattern may reflect a ceiling effect, as substantial IADL limitations constitute a core diagnostic criterion for dementia, thereby reducing this measure’s discriminative power at more advanced stages. In contrast, emerging functional difficulties may be more sensitive in capturing the transition to the prodromal MCI stage. Furthermore, the attenuated gradient observed for dementia may also be influenced by selective survival bias or differential clinical detection within the four-year observation window. In addition, the wider confidence intervals observed at the extremes of CES-D10 and IADL scores likely reflect a limited number of observations in these ranges, resulting in reduced precision of the predicted probabilities and warranting cautious interpretation.

The findings indicate that older age, multimorbidity, depressive symptoms, and IADL impairment were significantly associated with a higher likelihood of incident MCI and dementia. The attenuation of associations for BMI, grip strength, and lifestyle behaviors after multivariable adjustment likely reflects confounding by age, multimorbidity, and functional status, suggesting that these factors may operate indirectly through broader health and functional pathways rather than exerting independent effects. Multimorbidity has been consistently associated with more rapid cognitive decline and a higher risk of dementia across diverse populations [28], together with older age, depressive symptoms, and limitations in IADL [13,15,16]. Similarly, Asian population-based cohorts reported strong associations between multimorbidity and subsequent cognitive decline or incident MCI and dementia [29,30]. Evidence from prior multidomain intervention and observational studies further suggests that integrated risk profiles are meaningfully associated with cognitive trajectories [9,10,13,16].

In addition, greater social interaction was associated with a lower likelihood of dementia. These findings underscore the importance of preventive strategies targeting psychosocial and mental health factors, particularly depressive symptoms. The findings further highlight that living with family was associated with a lower risk for MCI and dementia. In the Korean sociocultural context, this robust protective effect—corresponding to a 69% and 72% lower relative risk—merits attention. Family co-residence in Korea traditionally functions as a multifunctional support system that provides instrumental assistance, emotional stimulation, and continuous monitoring of health-related changes. Such mechanisms may facilitate earlier recognition of subtle cognitive or behavioral changes and promote timely medical evaluation. However, several measurement-related considerations warrant a careful interpretation of these findings. In this study, living with family was operationalized as a binary indicator of co-residence (yes/no), which does not capture qualitative dimensions of family support, such as relationship quality, emotional closeness, caregiving burden, or familial strain. Similarly, social interaction was measured solely as the structural frequency of face-to-face contact. Although contact frequency represents an important dimension of social engagement, it does not capture qualitative aspects such as emotional closeness, reciprocity, or perceived support, which may also contribute to the maintenance of cognitive function in later life. Notably, the observed association between social interaction and dementia, but not MCI, may reflect stage-specific differences in the role of social connectedness across the cognitive decline continuum. Structural aspects of social engagement, such as contact frequency, may exert a more pronounced protective influence in later stages of cognitive impairment, whereas qualitatively rich, cognitively stimulating, and emotionally meaningful relationships may be more relevant during earlier, prodromal stages such as MCI. This interpretation remains speculative and should be examined in future studies using multidimensional measures of social relationships that capture both structural and qualitative components. Accordingly, the observed protective associations may reflect underlying perceived support or positive interpersonal interactions rather than structural exposure alone, warranting future research using more nuanced measures of family and social relationships. Overall, despite these measurement limitations, the present findings reinforce the importance of social and family support systems in the prevention of MCI and dementia [8,31,32]. Consistent with evidence from Asian population-based cohorts, including studies from China, Korea, and Japan, higher levels of social engagement have been associated with lower risks of cognitive decline and dementia [33,34,35,36].

For healthcare providers, these findings suggest integrating brief screening for these factors (e.g., IADL and depressive scales) into routine primary care to identify older adults with a higher risk for early preventive intervention. In Korea, where primary care and community health services are closely integrated with national screening programs, such multidomain risk stratification may be particularly feasible and scalable. Specifically, we suggest that the Korean National Health Screening Program for Elders could be strengthened by incorporating validated tools like the K-IADL and CES-D10 as low-cost, first-tier risk stratifiers. In practice, brief IADL and depression screening could be administered during routine primary care visits or community health check-ups, with individuals exceeding predefined risk thresholds referred for further cognitive evaluation or appropriate community-based support services. This risk stratification approach may also inform the prioritization of referrals to social prescribing initiatives, caregiver education, or community-based cognitive health programs, particularly for older adults living alone. Such tiered, low-burden approaches are well aligned with Korea’s primary care and public health infrastructure and may facilitate early identification and targeted prevention without substantial additional resource demands. The protective role of social interaction further underscores the need to strengthen community-based programs that promote social connectedness, reduce isolation, and support family- and community-centered aging environments [8,18,36]. Furthermore, large global prevention initiatives, including the World-Wide FINGERS consortium, have reinforced that integrating lifestyle, vascular, cognitive, and psychosocial components yields meaningful benefits for dementia prevention and healthy cognitive aging [37]. Consequently, integrating multidomain indicators into primary care and community health systems may enhance early identification and scalable, culturally appropriate preventive strategies.

## 5. Strengths and Limitations

This study has several strengths that enhance its relevance for informing community-based, culturally appropriate, and cost-effective dementia prevention strategies. First, the results are based on a large, nationally representative cohort with physician-diagnosed cognitive outcomes as reported by participants or proxies, which strengthens the validity and generalizability of the findings. Second, the multidomain analytic framework —integrating demographic, functional, psychosocial, and social factors—enabled a comprehensive assessment of baseline risk profiles relevant to cognitive decline in aging populations, thereby enhancing the robustness and interpretability of the results.

However, several limitations should be acknowledged. First, cognitive outcomes were defined based on self- or proxy-reported physician diagnoses rather than direct clinical assessments, which may be subject to underdiagnosis, misclassification, and differential detection. In addition, although physician diagnosis of MCI and dementia in South Korea is made within a standardized national health insurance system that requires adherence to uniform diagnostic criteria for reimbursement and service eligibility, the present study could not verify the specific diagnostic criteria applied at the individual level, reflecting an inherent limitation of survey-based data. Second, while chronic disease information was derived from physician-diagnosed reports, several other covariates—including hearing and vision problems and aspects of social interaction—relied on self- or proxy-reported data and may therefore be affected by measurement error or reporting bias [7,14]. Notably, social interaction was assessed using a single-item measure capturing only the frequency of face-to-face contact, without accounting for qualitative dimensions such as emotional closeness, reciprocity, or perceived support; thus, the observed associations should be interpreted as reflecting structural contact frequency rather than broader functional aspects of social connectedness. Third, despite the longitudinal design, the four-year follow-up period may be limited in showing longer-term trajectories or transitions across cognitive states, particularly considering the prolonged latency period of dementia. Fourth, although multiple domains were examined, several potentially important biological and lifestyle-related factors—including genetic susceptibility (e.g., APOE ε4 genotype), dietary quality (e.g., DASH or Mediterranean diet), sleep quality, medication use, and environmental exposures—were not available in the dataset and therefore could not be included in the analyses. These unmeasured factors may act as confounders or mediators and could partly explain or modify the observed associations; accordingly, the findings should be interpreted as associations rather than causal relationships. Finally, the relatively small number of incident MCI (n = 34) and dementia cases limited statistical power and resulted in low events-per-variable in the multinomial logistic regression models, potentially increasing the risk of coefficient instability, biased estimates, and inflated standard errors. Accordingly, estimates—particularly those with wide confidence intervals—should be interpreted with caution, and small cell sizes may limit the generalizability of specific findings to other populations or settings. Sensitivity analyses using a binary cognitive impairment outcome yielded directionally consistent results, supporting the robustness of the main findings. Additionally, because cognitive status was assessed at discrete survey waves, the analytic approach could not capture within-person transitions or the precise timing of cognitive decline onset; therefore, the findings should be interpreted as associations with incident cognitive status at follow-up rather than as estimates of disease progression dynamics.

## 6. Conclusions

The dose–response risk gradients generated from the adjusted probability analyses extend earlier work by offering absolute risk estimates that enhance clinical interpretability and support individualized assessment. Overall, these findings emphasize the global importance of multidomain approaches to understanding cognitive aging while offering culturally relevant insights specific to East Asian populations experiencing rapid demographic transitions. The present findings underscore the value of an evidence-based, multidomain risk profile that captures the concurrent contributions of demographic, functional, mental health, and social factors to cognitive decline. Importantly, this study demonstrates that multidomain risk assessment can be operationalized in primary care and community settings in Korea to facilitate early identification of older adults at elevated risk for MCI and dementia. Our results indicate that cognitive risk assessment should extend beyond age and cognitive testing to incorporate multidimensional markers, including multimorbidity, depressive symptoms, functional limitations, and social engagement. Embedding such multidomain screening within existing healthcare infrastructures—such as routine primary care visits and the Korean National Health Screening Program for older adults—may enable timely, targeted, and culturally congruent preventive strategies. In this context, social prescribing programs represent a promising translational pathway, linking high-risk older adults to community-based resources, social networks, and volunteer activities. When aligned with Korea’s strong tradition of family involvement in elder care, such approaches may further support family caregivers and mitigate social isolation—key modifiable contributors to cognitive decline. Taken together, this multidomain, evidence-based framework provides a practical foundation for dementia prevention strategies that integrate clinical screening with community and family-centered interventions in Korea.

## Figures and Tables

**Table 1:** Participants’ demographic characteristics in 2018 (n = 3432).

Variables	Sub-Categories	Normal Cognition (n = 3328)	MCI * (n = 34)	Dementia Diagnosis * (n = 70)	Differences *F* or *χ*^2^	*p*
		Mean ± SD or n (%)				
Age		74.20 ± 6.38	80.21 ± 4.93	81.19 ± 6.37	55.52	<0.001
Gender	Male	1373 (41.3%)	11 (32.4%)	22 (31.4%)	3.79	0.150
	Female	1955 (58.7%)	23 (67.6%)	48 (68.6%)		
Education level	Elementary school	1709 (51.4%)	27 (79.4%)	51 (72.9%)	24.52	<0.001
	Middle school	606 (18.2%)	4 (11.8%)	8 (11.4%)		
	High school	744 (22.4%)	2 (5.9%)	10 (14.3%)		
	College graduation or over	269 (8.1%)	1 (2.9%)	1 (1.4%)		
Living arrangement	living alone	642 (19.3%)	18 (52.9%)	32 (45.7%)	43.12	<0.001
	living with family	2686 (80.7%)	16 (47.1%)	38 (54.3%)		
Religion	Yes	1341 (40.3%)	13 (38.2%)	26 (37.1%)	0.34	0.844
	No	1987 (59.7%)	21 (61.8%)	44 (62.9%)		
Household income in the prior year (per 1000 KRW)		2289.05 ± 2563.19	1283.52 ± 1562.78	1812.89 ± 1975.13	3.70	0.025
BMI (kg/m^2^, per unit)		23.50 ± 2.69	22.00 ± 3.63	23.11 ± 2.63	5.43	0.004
Hand grip strength (kg)		23.69 ± 8.20	20.56 ± 7.98	19.05 ± 8.66	9.16	<0.001
Number of chronic diseases (count)		1.49 ± 1.19	2.09 ± 1.31	2.57 ± 1.64	31.35	<0.001
Depression (CES-D10)		6.34 ± 5.20	10.15 ± 3.96	15.79 ± 7.47	119.41	<0.001
Vision impairment	Yes	135 (4.1%)	3 (8.8%)	4 (5.7%)	2.35	0.310
	No	3180 (95.9%)	31 (91.2%)	66 (94.3%)		
Hearing impairment	Yes	88 (2.6%)	1 (2.9%)	5 (7.1%)	5.21	0.074
	No	3240 (97.4%)	33 (97.1%)	65 (92.9%)		
IADL score		11.05 ± 4.41	15.12 ± 10.40	16.43 ± 12.09	54.98	<0.001
Drinking	None	1776 (53.4%)	21 (61.8%)	44 (62.9%)	7.78	0.100
	Past	651 (19.6%)	9 (26.5%)	14 (20.0%)		
	Current	900 (27.1%)	4 (11.8%)	12 (17.1%)		
Smoking	None	2340 (70.7%)	27 (79.4%)	51 (73.9%)	5.58	0.232
	Past	735 (22.2%)	4 (11.8%)	17 (24.6%)		
	Current	237 (7.2%)	3 (8.8%)	1 (1.4%)		
Regular exercise	Yes	1158 (34.8%)	5 (14.7%)	13 (18.6%)	13.84	<0.001
	No	2170 (65.2%)	29 (85.3%)	57 (81.4%)		
Social interactions (10-point contact frequency)		7.28 ± 2.70	6.74 ± 3.24	3.16 ± 3.51	79.05	<0.001

Notes: * 2022 diagnosis. Abbreviations: MCI = mild cognitive impairment; IADL = instrumental activities of daily living; CES-D10 = 10-item Center for Epidemiologic Studies Depression scale; KRW = Korean won.

**Table 2:** Multivariable predictors of 4-year incident mild cognitive impairment and dementia.

Variables	MCI (n = 34)			Dementia (n = 70)		
	RRR	CI (95%)	*p*	RRR	CI (95%)	*p*
Age (per year)	1.10	1.02–1.18	0.014	1.12	1.05–1.19	<0.001
Gender (ref: Male)	0.62	0.15–2.53	0.500	1.31	0.39–4.42	0.668
Education level (ref: Elementary school)						
Middle school	1.38	0.41–4.70	0.601	0.63	0.19–2.09	0.450
High school	0.69	0.14–3.55	0.659	1.14	0.38–3.40	0.815
College or higher	0.78	0.08–7.25	0.825	0.27	0.02–3.53	0.321
Living arrangement (ref: living alone)						
living with family	0.31	0.11–0.85	0.023	0.28	0.12–0.64	0.003
Religion (ref. No)	1.75	0.72–4.24	0.216	1.52	0.71–3.23	0.283
Household income in the prior year (per 1000 KRW)	1.00	1.00–1.00	0.425	1.00	1.00–1.00	0.957
BMI (kg/m^2^, per unit)	0.89	0.77–1.04	0.141	0.96	0.85–1.08	0.498
Hand grip strength (kg)	1.02	0.95–1.09	0.627	1.01	0.96–1.07	0.645
Number of chronic diseases (count)	1.17	0.82–1.66	0.382	1.48	1.11–1.97	0.008
Depressive symptoms (CES-D10) (30-point score)	1.09	1.01–1.18	0.032	1.24	1.16–1.32	<0.001
Vision impairment (ref. No)	1.34	0.29–6.21	0.705	0.18	0.02–1.83	0.146
Hearing impairment (ref. No)	1.17	0.13–10.33	0.890	2.92	0.70–12.10	0.140
IADL score	1.11	1.06–1.17	<0.001	1.07	1.02–1.13	0.012
Drinking (ref. Current)						
Past	2.17	0.51–9.17	0.293	0.40	0.13–1.23	0.109
Never	1.08	0.26–4.58	0.915	0.45	0.16–1.28	0.133
Smoking (ref. Never)						
Past	0.37	0.08–1.71	0.205	1.33	0.43–4.15	0.618
Current	0.50	0.06–4.07	0.517	0.35	0.04–3.24	0.354
Regular exercise (ref. No)	0.84	0.29–2.46	0.746	1.54	0.67–3.55	0.309
Social interaction	0.97	0.84–1.13	0.741	0.72	0.63–0.81	<0.001
(10-point contact frequency)						
	Prob > chi^2^ ≤ 0.001			Prob > chi^2^ ≤ 0.001		

Notes: Values are relative risk ratios (RRRs), 95% confidence intervals (CIs), and p-values from multinomial logistic regression (reference outcome: normal cognition). Household income is scaled per 1000 KRW. Abbreviations: MCI = mild cognitive impairment; IADL = instrumental activities of daily living; CES-D10 = 10-item Center for Epidemiologic Studies Depression scale; KRW = Korean won.

## Data Availability

The data that support the findings of this study are third-party data from the Korean Longitudinal Study of Aging (KLoSA), administered by Statistics Korea. The de-identified KLoSA datasets are publicly available for scientific research and can be accessed upon reasonable request and approval via the KLoSA data portal (https://survey.keis.or.kr/eng/klosa/klosa01.jsp (accessed on 18 September 2025)).
